# Formyl Peptide Receptor 1 Modulates Endothelial Cell Functions by NADPH Oxidase-Dependent VEGFR2 Transactivation

**DOI:** 10.1155/2018/2609847

**Published:** 2018-03-18

**Authors:** Fabio Cattaneo, Martina Castaldo, Melania Parisi, Raffaella Faraonio, Gabriella Esposito, Rosario Ammendola

**Affiliations:** Department of Molecular Medicine and Medical Biotechnology, School of Medicine, University of Naples Federico II, Naples, Italy

## Abstract

In the vasculature, NADPH oxidase is the main contributor of reactive oxygen species (ROS) which play a key role in endothelial signalling and functions. We demonstrate that ECV304 cells express p47^phox^, p67^phox^, and p22^phox^ subunits of NADPH oxidase, as well as formyl peptide receptors 1 and 3 (FPR1/3), which are members of the GPCR family. By RT-PCR, we also detected Flt-1 and Flk-1/KDR in these cells. Stimulation of FPR1 by N-fMLP induces p47^phox^ phosphorylation, which is the crucial event for NADPH oxidase-dependent superoxide production. Transphosphorylation of RTKs by GPCRs is a biological mechanism through which the information exchange is amplified throughout the cell. ROS act as signalling intermediates in the transactivation mechanism. We show that N-fMLP stimulation induces the phosphorylation of cytosolic Y951, Y996, and Y1175 residues of VEGFR2, which constitute the anchoring sites for signalling molecules. These, in turn, activate PI3K/Akt and PLC-*γ*1/PKC intracellular pathways. FPR1-induced ROS production plays a critical role in this cross-talk mechanism. In fact, inhibition of FPR1 and/or NADPH oxidase functions prevents VEGFR2 transactivation and the triggering of the downstream signalling cascades. N-fMLP stimulation also ameliorates cellular migration and capillary-like network formation ability of ECV304 cells.

## 1. Introduction

Vascular endothelial growth factor receptor 2 (VEGFR2)/Flk-1/KDR and VEGFR1/Flt1 are members of the receptor tyrosine kinase (RTK) family and bind to vascular endothelial growth factor (VEGF) promoting organization, migration, proliferation, and formation of vascular structures of endothelial cells (ECs) [1]. In the human VEGFR2, Y951, Y1054, Y1059, Y1175, and Y1214 residues have been detected as phosphorylation sites [2, 3] and Y801, Y996, and Y1008 residues have been involved in VEGFR2 signalling [4, 5]. The phosphorylated Y1175 residue binds to phospholipase C*γ* (PLC-*γ*) [3], as well as with the adaptor molecules Shb [6] and Sck [7], whereas the phosphorylated Y951 residue mediates binding for VEGF receptor-associated protein (VRAP), which is also known as a T cell-specific adapter (TSAd), which is crucial for EC migration in vitro and cell actin reorganization [2]. The phosphorylated Y1214 residue of VEGFR2 represents an anchoring site for the adaptor protein Nck [8], whereas the role of the phosphorylation of Y1224, Y1305, Y1309, and Y1319 residues in the C-terminal tail still remains to be determined.

The G protein-coupled receptors (GPCRs) are a superfamily of plasma membrane proteins activated by several ligands. Their agonist-specific stimulation induces G protein dissociation and, in turn, the activation of membrane-associated enzymes, intracellular second messengers, or ion channels. The human formyl peptide receptors 1, 2, and 3 (FPR1, FPR2, and FPR3) are members of the GPCR family and are all associated with pertussis toxin- (PTX-) sensitive Gi proteins [9–11]. FPR1 binds to efficiently N-formyl-methionyl-leucyl-phenylalanine (N-fMLP), whereas FPR2 is effectively activated by low concentrations of WKYMVm peptide [12]. The significant biological functions of FPR1 and FPR2 are supported by the discovery of high-affinity host-derived ligands. These two receptors are expressed in several cell types [13, 14], whereas FPR3, which does not bind to N-fMLP or WKYMVm, is expressed in monocytes, dendritic cells [9–11], and human umbilical vein endothelial cell (HUVEC) primary cultures [15]. FPR2 is also expressed on nuclear membranes of human lung carcinoma CaLu-6 and human gastric adenocarcinoma AGS cell lines [16, 17].

The most important source of ROS in ECs is NADPH oxidase, which consists of cytosolic subunits p47^phox^, p40^phox^, p67^phox^, and the small GTPase Rac1 and of membrane-associated proteins p22^phox^ and gp91^phox^. In several cell types, FPR stimulation by N-fMLP or WKYMVm induces superoxide generation as a consequence of MEK- and PKC-dependent phosphorylation of the regulatory subunit p47^phox^, which is in large part prevented by preincubation with PTX [13, 18–20]. NADPH oxidase-derived ROS act as intracellular second messengers by activating several redox signalling cascades implicated in VEGFR2 autophosphorylation, EC migration, angiogenesis, proliferation [21], and postnatal angiogenesis *in vivo* [22]. Nevertheless, molecular mechanisms responsible for NADPH oxidase activation and the function of ROS in redox signalling linked to angiogenesis remain unclear.

Even though GPCRs are deficient of an intrinsic tyrosine kinase activity, binding of specific ligands may induce tyrosine phosphorylation of RTKs. The agonist-dependent stimulation of GPCRs can enhance the signalling activity of RTKs, linking the ample heterogeneity of GPCRs with the effective signalling abilities of RTKs. Transactivation of RTKs by GPCRs may occur by diverse molecular mechanisms, which include the activation of metalloexopeptidases and metalloendopeptidases, the involvement of nonreceptor tyrosine kinases associated with the membrane, or NADPH oxidase-dependent ROS generation [23]. In different cell types, FPR2 stimulation prompts phosphorylation of tyrosine residues of EGFR, which provide anchoring sites for the recruitment and activation of intracellular signalling pathways [24], and HGF receptor transphosphorylation, thereby inducing part of the molecular responses triggered by c-Met/HGF binding [19]. ROS play a crucial role in these cross-talk mechanisms since the inhibition of NADPH oxidase functions prevents EGFR and c-Met transactivation [19, 24].

Herein, we show that ECV304 cells express FPR1, Flk-1/KDR, and p47^phox^ and that FPR1 stimulation by N-fMLP induces NADPH oxidase-dependent ROS generation as well as the transphosphorylation of cytosolic Y951, Y996, and Y1175 residues of VEGFR2. These phosphotyrosines represent anchoring sites for signalling molecules that, in turn, activate PI3K/Akt and PLC-*γ*1/PKC intracellular pathways involved in cell attachment and cell migration of ECs. Furthermore, FPR1 activation also ameliorates cellular migration and capillary-like network formation of ECV304 cells.

## 2. Materials and Methods

### 2.1. Antibodies and Chemicals

The N-fMLP peptide was synthesized and HPLC-purified by PRIMM (Milan, Italy). SDS-PAGE reagents were purchased from Bio-Rad (Hercules, CA, USA). Protein A/G Plus, anti-Flk1, anti-p-Flk1 (Tyr951), anti-p-Flk1 (Tyr996), anti-p-Flk1 (Tyr1175), anti-p-Flk1 (Tyr1214), anti-p-Tyr, anti-p47^phox^, anti-p22^phox^, anti-p-PLC*γ*1 (Y783), anti-PLC*γ*1, anti-PKC*α*, anti-PKC*β*II, anti-PKC*ζ*, anti-PKC*δ*, anti-tubulin, anti-mouse, and anti-rabbit were from Santa Cruz Biotechnology (Santa Cruz, CA, USA). Anti-p-PI3K (p85) and anti-p-Akt (Ser473) were from Cell Signaling Technology (Danvers, MA, USA). Anti-p-Ser, p22^phox^ siRNA (SI03078523), and scramble control siRNA (SI03650318) were from Qiagen (Hiden, Germany). FPR1 siRNA (L-005140-00) and scramble control (D-001810-10) were purchased from Dharmacon (Lafayette, CO, USA). Protein A-horseradish peroxidase was from Amersham Pharmacia Biotech (Little Chalfont, Buckinghamshire, UK). Pertussis toxin (PTX), apocynin, wortmannin, and LY294002 were from Sigma (St. Louis, MO, USA).

### 2.2. RNA Purification and RT-PCR Analysis

Total RNA was purified from ECV304 cells by TRIzol reagents (Thermo Fisher Scientific) according to the manufacturer's instruction, and 0.1 *μ*g of RNA was used as a template for reverse transcription experiments, as previously described [25]. Primer sequences designed to amplify human coding regions and relative product sizes are reported in Table 1.

### 2.3. Cell Culture

ECV304 cells (ATCC®CRL-1998) were obtained from ATCC (Rockville, MD, USA) and were grown in Dulbecco's modified Eagle's medium (DMEM) containing 10% fetal bovine serum (FBS), 100 U/ml penicillin, and 100 *μ*g/ml streptomycin. Cells were serum-starved for 24 hours, once they reached 80% of confluence, and then stimulated with 0.1 *μ*M N-fMLP peptide for various times, as reported in the figures. In other experiments, serum-starved cells were preincubated with 100 ng/ml PTX for 16 hours, 50 *μ*M LY294002 for 1 hour, 0.5 *μ*M wortmannin for 1 hour, or 100 *μ*M apocynin for 2 hours, before stimulation with 0.1 *μ*M N-fMLP for 5 minutes. Short interfering RNA experiments were performed incubating 4 × 10^5^ cells with 5 nM siRNAs for 12 hours, in DMEM containing 10% FBS and 20 *μ*l of HiPerFect (Qiagen, Hiden, Germany). Cells were then serum-starved for 24 hours and stimulated with 0.1 *μ*M N-fMLP for 5 minutes. ECV304 cells were also incubated with 20 ng/ml VEGF, as a control.

### 2.4. Western Blotting and Immunoprecipitation Assays

ECV304 cells were incubated with N-fMLP with or without specific inhibitors as described above. Whole lysates were purified in buffer containing 150 mmol/l NaCl, 50 mmol/l Tris-HCl (pH 8.5), 2 mmol/l EDTA, 1% *v*/*v* NP-40, 0.5% *w*/*v* deoxycholate, 10 mmol/l NaF, 10 mM sodium pyrophosphate, 2 mmol/l PMSF, 2 *μ*g/ml leupeptin, and 2 *μ*g/ml aprotinin (pH 7.4), as previously described [26]. Lysates were incubated at 0°C for 15 min and then centrifuged at 38000 ×g for 15 min at 4°C. Purification of membrane proteins was performed as previously described [19]. Bio-Rad protein assay (Bio-Rad, Hercules, CA, USA) was used to determine protein concentration. Proteins were resolved on a 10% SDS-PAGE, and immunoblot experiments were performed as previously described [24]. Immunoprecipitation experiments were performed by incubating equal amounts of proteins with 3 *μ*g of anti-p47^phox^ or anti-Flk1 antibodies. Protein expression or phosphorylation was detected by the ECL chemiluminescence reagent kit (Amersham Pharmacia Biotech) and visualized by autoradiography. Densitometry analysis was used to quantify protein or phosphorylation levels by using a Discover Pharmacia scanner.

### 2.5. Superoxide Production Assay

Membranes and cytosol fractions were purified from serum-starved ECV304 cells stimulated with 0.1 *μ*M N-fMLP for the times reported in the figure. The reduction of cytochrome c was measured to determine NADPH-dependent superoxide generation, as previously described [24]. Briefly, 10 *μ*g of membrane proteins and 200 *μ*g of cytosolic proteins were incubated in PBS in the presence of 15 *μ*M GTP-*γ*-S, 100 *μ*M cytochrome c, and 10 *μ*M FAD in a final volume of 1 ml. Superoxide production was monitored at 550 nm, after the addition of 100 *μ*M NADPH. Cells were also incubated with 200 U/ml superoxide dismutase (SOD), as the control of the specificity of cytochrome c reduction. Superoxide anion generation was measured as the SOD-inhibitable reduction of ferricytochrome c. Individual treatments were compared with the values obtained from growth-arrested ECV304 cells by Student's *t*-test.

### 2.6. Cell Migration Assay

ECV304 cells were grown as described above until they reached 100% confluence, and a wound was induced in the monolayer by scratching it with a sterile 80 *μ*m diameter tip. Cells were incubated in serum-deprived medium at 37°C at 5% CO_2_, and time-lapse images were taken every 12 hours up to 36 hours after wound generation by using the Leica AF6000 Modular System and processed by using the Leica LAS AF light software. The covered surface was quantified with the ImageJ software.

### 2.7. Capillary-Like Network Formation

48-multiwell plates were coated with 150 *μ*L of Matrigel (BD Bioscience) per well and then allowed to polymerize for 30 minutes at 37°C, according to the manufacturer's instructions. 1 × 10^5^ ECV304 cells, pretreated or not with 100 ng/ml PTX, were plated in the precoated wells with serum-free DMEM in the presence or absence of 0.1 *μ*M N-fMLP or 20 ng/ml VEGF (Gold Biotechnology, Olivette, USA) for 16 hours at 37°C. Network formation was acquired with the Leica AF6000 Modular System, and the total tube length was measured by using the ImageJ software.

### 2.8. Statistical Analysis

All the reported data are expressed as means ± SD and represent at least three unrelated experiments. Statistical analyses were evaluated by Student's *t*-test, and their significance was considered with a minimum value of *p* < 0.05. All statistical analyses were performed with the Prism statistical software.

## 3. Results and Discussion

### 3.1. ECV304 Cells Express Flt-1, Flk-1/KDR, NADPH Oxidase, and a Functional FPR1 Receptor

ECV304 cells were initially described as a HUVEC-derived transformed cell line [27], but they were later characterized, by genetic relationship, as a cell line derived from human urinary bladder carcinoma T24 cells [28]. Nevertheless, although not of HUVEC origin, ECV304 cells show many characteristics of ECs [29, 30] and present both epithelial and endothelial features [31], a number of which are solely endothelial markers and consequently not detected in T24 cells [30]. Therefore, ECV304 cells seem to be a relevant model for the study of molecular mechanisms in the endothelium, such as signal transduction, cell migration, and capillary-like network formation.

**Figure 1:**
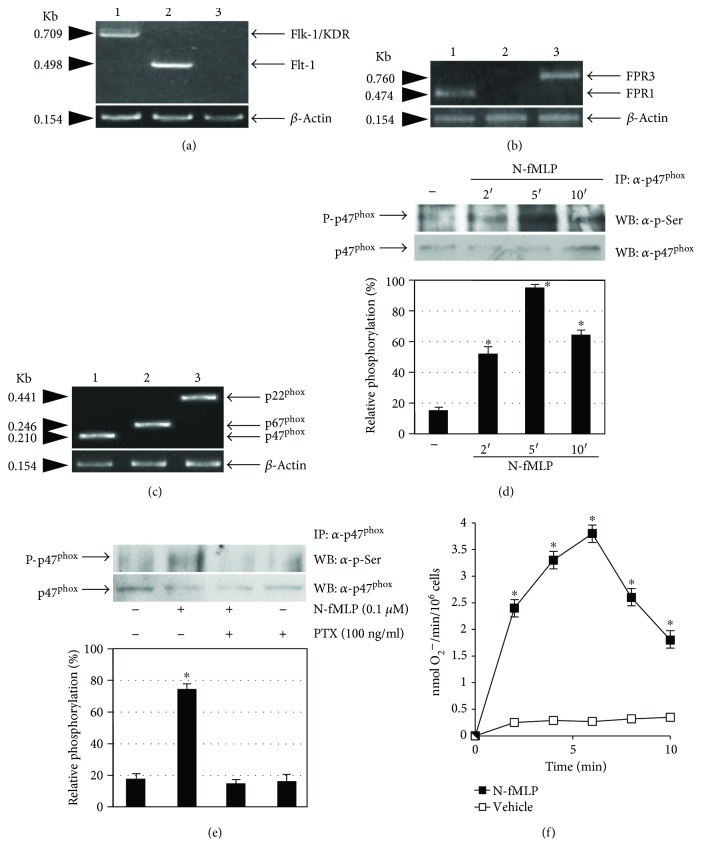
ECV304 cells express Flk-1/KDR, FPR1, and NADPH oxidase. Total RNA was purified from ECV304 cells. cDNA was coamplified by using (a) Flk-1/KDR (lane 1), Flt-1 (lane 2), or Flt-4 (lane 3); (b) FPR1 (lane 1), FPR2 (lane 2), or FPR3 (lane 3); and (c) p47^phox^ (lane 1), p67^phox^ (lane 2), p22^phox^ (lane 3), or *β*-actin primers. PCR products were separated on a 1.5% agarose gel and stained with ethidium bromide. (d) Serum-starved ECV304 cells were stimulated with 0.1 *μ*M N-fMLP for the indicated times or (e) preincubated with PTX before N-fMLP stimulation for 5 minutes. Whole lysates (1 mg) were immunoprecipitated with an *α*-p47^phox^ antibody and resolved on 10% SDS-PAGE. p47^phox^ phosphorylation was determined by using an *α*-p-Ser antibody. An *α*-p47^phox^ antibody served as a control for protein loading. (f) Superoxide production was determined as a SOD-sensitive rate reduction of cytochrome c in serum-starved ECV304 cells stimulated or not with 0.1 *μ*M N-fMLP for the indicated times. All the experiments are representative of at least three independent experiments. ^∗^*p* < 0.05 compared with unstimulated cells.

In these cells, we detected, by RT-PCR, the expression of Flt-1 and Flk-1/KDR (Figure 1(a)), but not of Flt-4, which is expressed only in lymphatic endothelial cells. The two VEGF receptors were also detected by immunostaining in ECV304 cells [32]. We also analyzed the expression of FPRs, and we provided the first evidence that FPR1 and FPR3 but not FPR2 (Figure 1(b)) are expressed in ECV304 cells. This cell line also expresses p47^phox^, p67^phox^, and p22^phox^ subunits of the NADPH oxidase enzymatic complex (Figure 1(c)). In human fibroblasts, stimulation of FPRs with N-fMLP induces p47^phox^ phosphorylation, which is the crucial event required for NADPH oxidase activation [13]. In ECV304 cells, FPR1 is a functional receptor; in fact, stimulation with 0.1 *μ*M N-fMLP triggers time-dependent phosphorylation of p47^phox^ (Figure 1(d)) which is entirely inhibited by preincubation with PTX (Figure 1(e)). Furthermore, incubation with N-fMLP for different times stimulates NADPH oxidase-dependent superoxide production, with a maximum of ROS generation occurring at 6 min (Figure 1(f)).

### 3.2. FPR1 Stimulation by N-fMLP Promotes Flk-1/KDR Transactivation

Cellular effects of VEGF-A on ECs, such as permeability, migration, survival, and proliferation, are mediated by Flk-1/KDR, which binds VEGF-A to the second and third extracellular Ig-like domains. This allows the correct placement of the intracellular kinase domains, which results in the Flk-1/KDR autophosphorylation [33].

**Figure 2:**
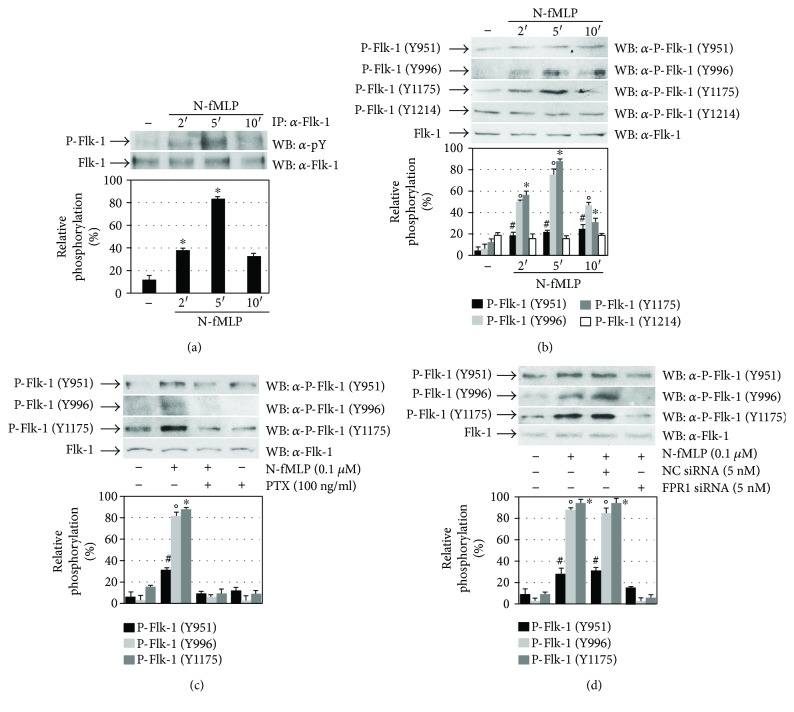
FPR1 activation promotes Flk-1/KDR transphosphorylation. (a) Whole lysates (900 *μ*g) purified from serum-starved ECV304 cells stimulated with 0.1 *μ*M N-fMLP for the indicated times were immunoprecipitated with an *α*-Flk-1 antibody, and Flk-1 tyrosine phosphorylation was detected with an *α*-pY antibody. An *α*-Flk1 antibody served as a control for protein loading. (b, c, d) Fifty micrograms of whole lysates, purified from ECV304 cells, was resolved on 10% SDS-PAGE. The cells were (b) stimulated with N-fMLP for the indicated times, (c) stimulated with N-fMLP for 5 minutes in the presence or absence of PTX, or (d) incubated for 12 hours with 5 nM siRNA against FPR1 (FPR1 siRNA) or negative control siRNA (NC siRNA) in DMEM containing 10% FBS in the presence of 20 *μ*l of HiPerfect. The filters were immunoblotted with *α*-pFlk-1 (Y951), *α*-pFlk-1 (Y996), *α*-pFlk-1 (Y1175), or *α*-pFlk-1 (Y1214) antibodies. An *α*-Flk-1 antibody served as a control for protein loading. ^∗^*p* < 0.05 compared with unstimulated cells. °*p* < 0.05 compared with unstimulated cells. ^#^*p* < 0.05 compared with unstimulated cells.

Cross-talk between GPCRs and RTKs modulates downstream signalling pathways involved in many biological functions of mammalian cells [19, 23, 24, 34]. Therefore, we analyzed Flk-1/KDR transactivation by FPR1 in ECV304 cells, and in Western blot experiments, we noticed that the incubation with 0.1 *μ*M N-fMLP increases Flk-1/KDR tyrosine phosphorylation in a time-dependent manner (Figure 2(a)). VEGFR2 is the main signal transducer in ECs, and in the human Flk-1/KDR intracellular domain, multiple tyrosine residues have been detected as phosphorylation sites, including Y801, Y951, Y996, Y1008, Y1054, Y1059, Y1175, and Y1214 [2–4]. The FPR1 agonist triggers the phosphorylation of Y951, Y996, and Y1175 residues of Flk-1/KDR within the first 5 min (Figure 2(b)), which is completely inhibited by preincubating ECV304 cells with PTX before N-fMLP exposure (Figure 2(c)). A significant reduction in the phosphorylation levels of Y951, Y996, and Y1175 residues is observed when cells are preincubated with siRNAs against FPR1 before N-fMLP treatment (Figure 2(d)), indicating that VEGFR2 transphosphorylation is mediated by FPR1.

### 3.3. Flk-1/KDR Transactivation Depends on NADPH Oxidase-Dependent ROS Generation

**Figure 3:**
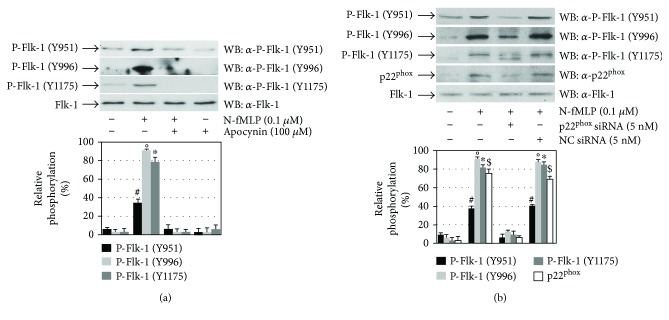
Flk-1/KDR transactivation depends on NADPH oxidase activation. (a) Serum-starved ECV304 cells were stimulated with 0.1 *μ*M N-fMLP for 5 minutes in the presence or absence of 100 *μ*M apocynin, or (b) ECV304 cells serum-deprived for 24 hours were incubated for 12 hours with 5 nM siRNA against p22^phox^ (p22^phox^ siRNA) or negative control siRNA (NC siRNA) in DMEM containing 10% FBS in the presence of 20 *μ*l of HiPerfect and stimulated with 0.1 *μ*M N-fMLP for 5 minutes. Fifty micrograms of whole lysates were resolved on 10% SDS-PAGE and immunoblotted with *α*-pFlk-1 (Y951), *α*-pFlk-1 (Y996), *α*-pFlk-1 (Y1175), *α*-pFlk-1 (Y1214), or *α*-p22phox antibodies. An *α*-Flk-1 antibody served as a control for protein loading. All the experiments are representative of at least three independent experiments. ^∗^*p* < 0.05 compared with unstimulated cells. °*p* < 0.05 compared with unstimulated cells. ^#^*p* < 0.05 compared with unstimulated cells. ^$^*p* < 0.05 compared with unstimulated cells.

The main source of ROS in the arterial wall and ECs is NADPH oxidase [35], which can be activated by several stimuli including GPCR agonists [23, 36]. In nonphagocytic cells, NADPH oxidase expression depends on the cellular types and surrounding conditions and produces ROS at low levels [21, 23], which can act as signalling molecules by reversible oxidation/reduction of cysteines located in the catalytic site of protein tyrosine phosphatases (PTPs) [19, 21, 37, 38]. ROS can play a role in RTK transphosphorylation by preventing the PTPase action and, in turn, changing the RTK from a nonphosphorylated to a phosphorylated state. A number of PTPs, such as LMW-PTP (HCPTPA), SHP-1, and SHP-2, are associated with Flk-1/KDR upon VEGF stimulation [39, 40]. We preincubated ECV304 cells with apocynin (Figure 3(a)), which specifically inhibits NADPH oxidase, or with a siRNA against p22^phox^ (Figure 3(b)), an essential component of the membrane-associated NADPH oxidase, and we noticed that the FPR1-induced transphosphorylation of Y951, Y996, and Y1175 residues of Flk-1/KDR is prevented by the arrest of NADPH oxidase functions (Figures 3(a) and 3(b)). These results demonstrate that FPR1-mediated superoxide generation feeds the cross-talk between FPR1 and Flk-1/KDR.

### 3.4. FPR1-Induced Flk-1/KDR Transactivation Triggers the PI3K/Akt Pathway

Phosphorylated tyrosine residues of Flk-1/KDR represent anchoring sites for signalling molecules that trigger intracellular pathways, which, in turn, activate biological responses such as cell proliferation and migration [41]. In the human VEGFR2, several phospho-tyrosines have been identified [1–4] and so far Y951, Y996, Y1054, Y1059, Y1175, and Y1214, in the kinase insert domain, in the kinase domain, and in the C-terminal tail of VEGFR2, have been defined as the main autophosphorylation sites [42]. Phosphorylation of Y1175, within the pYIVL sequence, provides a docking site for several signalling molecules, including PLC-*γ* [3] and the adaptor proteins Sck [7] and Shb [6]. Shb contains an SH2 and a PTB domain, four presumed tyrosine phosphorylation sites, and a proline-rich N-terminus motif [6]. Shb binds to phospho-tyrosine Y1175 of VEGFR2, resulting in its Src-dependent phosphorylation [6]. Shb-dependent binding to the Y1175 residue is important for the PI3K response, but it is unclear how this effect is pursued. The SH3 domain of PI3K (p85) could interact with Shb at the level of the proline-rich motif; otherwise, the effect could be mediated by focal adhesion kinase (FAK), which is involved in cellular attachment and migration [43]. Silencing of Shb by small interfering RNA (siRNA) results in the arrest of PI3K activation.

The phosphorylated Y951 residue in the VEGFR2 kinase insert domain binds to TSAd which is equivalent to Rlk- and Itk-binding protein (RIBP), Lck adaptor (LAD), and VRAP [44]. Y951-mediated binding between VEGFR2 and TSAd plays a critical role in cell migration of ECs and VEGF-induced actin reorganization. In fact, site-directed mutagenesis of Y951 to F951 in Flk1/KDR, or silencing by siRNA of VRAP/TSAd expression, prevents VEGFA-mediated migration [2]. Stimulated RTKs typically activate PI3K by inducing phosphorylation of a tyrosine residue within an YXXM motif, which represents an anchoring site for SH2 domains of the p85 regulatory subunit of PI3K. Binding of PI3K to the YXXM motif mediates Akt activation. Phosphorylation of the tyrosine residue is mediated by TSAd, which activates members of Src family kinases [45, 46]. Flk-1/KDR does not have a pYXXM motif detected by the SH2 domain of the p85 subunit [47]; nevertheless, a binding site for the p85 subunit of PI3K is localized in the Gab1 adaptor protein, which also binds to VEGFR2, although the exact binding site in the receptor is unknown [48, 49].

**Figure 4:**
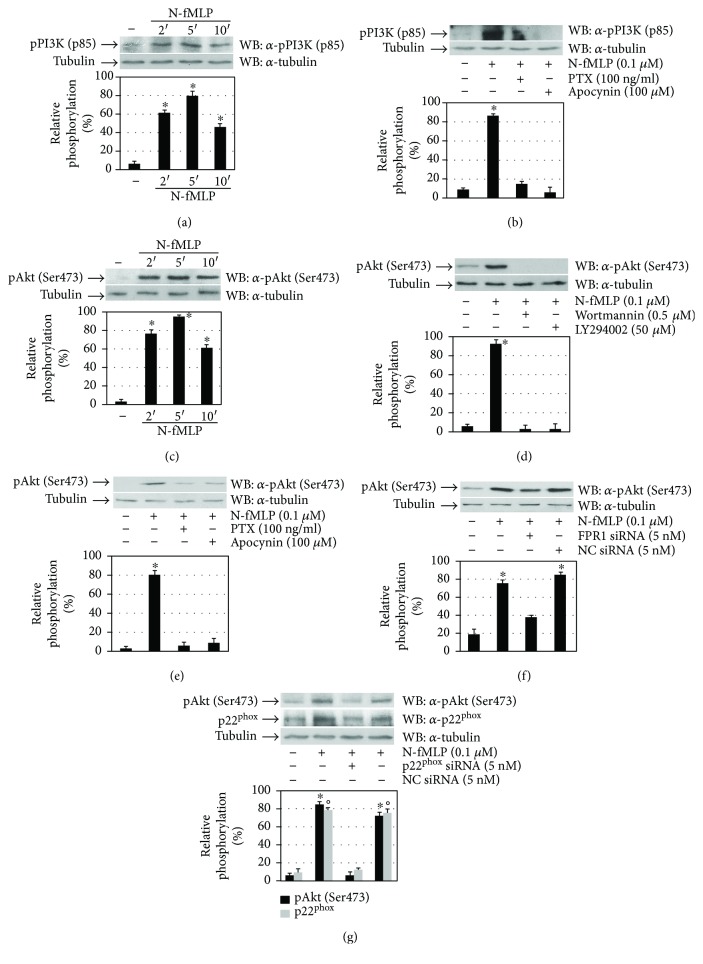
FPR1-mediated Flk-1/KDR transactivation triggers the PI3K-Akt pathway. (a, c) Cell lysates were purified from serum-starved ECV304 cells exposed to 0.1 *μ*M N-fMLP for the indicated times. (b, e) ECV304 cells were serum-deprived for 24 hours before the stimulation for 5 minutes with N-fMLP in the absence or presence of PTX, apocynin, (d) wortmannin, or LY294002. (f) ECV304 cells were serum-deprived for 24 hours, incubated for 12 hours with 5 nM siRNA against FPR1 (FPR1 siRNA) or (g) against p22^phox^ (p22^phox^ siRNA) or negative control siRNA (NC siRNA), in DMEM containing 10% FBS in the presence of 20 *μ*l of HiPerfect, and stimulated with 0.1 *μ*M N-fMLP for 5 minutes. Fifty micrograms of whole lysates was resolved on 10% SDS-PAGE and immunoblotted with (a, b) an *α*-pPI3K (p85) or (c–g) an *α*-pAkt (S473) antibody. An *α*-tubulin antibody served as a control for protein loading. All the experiments are representative of at least three independent experiments. ^∗^*p* < 0.05 compared with unstimulated cells. °*p* < 0.05 compared with unstimulated cells.

In ECs, VEGFA-induced cell survival depends on Flk-1/KDR and on the consequent activation of PI3K and Akt, which induces A1 and Bcl-2 expression [50]. We analyzed PI3K activation in N-fMLP-stimulated ECV3014 cells, and in Western blot experiments, we detected that N-fMLP stimulates PI3K (p85) phosphorylation in a time-dependent manner (Figure 4(a)). This is prevented by pretreating EVC304 cells with PTX or apocynin (Figure 4(b)), suggesting that PI3K (p85) phosphorylation depends on PTX-sensible GPCR and NADPH oxidase-dependent superoxide generation.

Activation of PI3K and production of phosphatidylinositol (3,4,5)-trisphosphate (PIP3) result in the consequent activation of Akt by PDK1 and PDK2, which phosphorylate Akt at T308 and S473 residues, respectively. We analyzed Akt phosphorylation in FPR1-stimulated ECV304 cells, and we observed that N-fMLP induces Akt (S473) phosphorylation in the same time interval of PI3K (p85) phosphorylation (Figure 4(c)). Akt (S473) phosphorylation is prevented by preincubation of ECV304 cells with selective PI3K inhibitors (Figure 4(d)), as well as with PTX, which blocks FPR1-bound G_i_ proteins in their inactive form, or with apocynin (Figure 4(e)). The critical role of FPR1 and NADPH oxidase in the Akt (Ser473) phosphorylation is supported by the finding that preincubation with siRNAs against FPR1 (Figure 4(f)) or against p22^phox^ (Figure 4(g)) before N-fMLP treatment results in a substantial decrease in the phosphorylation levels of Akt.

### 3.5. FPR1-Mediated Phosphorylation of the Y1175 Residue Provides a Docking Site for PLC-*γ*1 Activation

The phosphorylated Y1175 residue of Flk-1/KDR represents a binding site for PLC-*γ*1 [3] and other adaptor proteins [6, 7]. PLC-*γ*1 is phosphorylated and its catalytic activity is enhanced as a consequence of binding to pY1175. PLC-*γ* plays a crucial role in angiogenesis, as demonstrated by the observation that PLC-*γ*1-deficient mouse embryos die at nearly E9.0 with substantially reduced erythropoiesis and vasculogenesis [51, 52] and that the mutation of Y1173 in mice (Y1175 in human) is responsible for the embryonic mortality at E8.5–9.5, as a consequence of anomalies of haematopoietic and endothelial cells [53]. Furthermore, in zebrafish, PLC-*γ*1 is required for arterial development, as demonstrated by the observation that zebrafish embryos lacking in PLC-*γ*1 do not respond to VEGF [54]. These results support the idea that signalling from pY1175 of VEGFR2 to the PLC-*γ*/PKC pathway is essential for vasculogenesis in embryogenesis.

**Figure 5:**
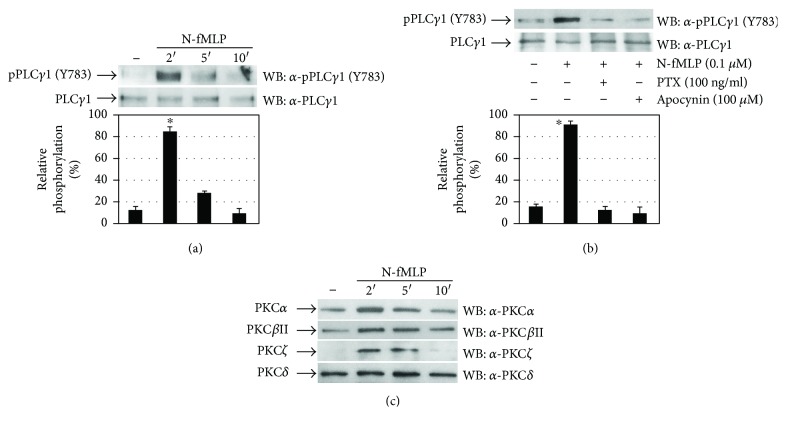
Tyrosine phosphorylation of 1175 residues of Flk-1/KDR triggers the PLC-*γ*1/PKC pathway. (a, c) Serum-deprived ECV304 cells were stimulated with 0.1 *μ*M N-fMLP for the indicated times or (b) for 5 minutes in the presence or absence of PTX or apocynin. (a, b) Fifty micrograms of whole lysates was resolved on 10% SDS-PAGE, and PLC*γ*1 phosphorylation on the Y783 residue was detected by using an *α*-pPLC*γ*1 (Y783) antibody. An *α*-PLC*γ*1 antibody was used as a control for protein loading. (c) Membrane proteins (50 *μ*g) were resolved on 10% SDS-PAGE, and PKC isoforms were detected by using the specific antibodies *α*-PKC*α*, *α*-PKC*β*II, *α*-PKC*ζ*, or *α*-PKC*δ* as indicated. All the experiments are representative of at least three independent experiments. ^∗^*p* < 0.05 compared with unstimulated cells.

Four activation-induced tyrosine phosphorylation sites (Y472, Y771, Y783, and Y1254) have been described in PLC-*γ*1 [55]. In time-course experiments, we observed that N-fMLP induces PLC-*γ*1 activation with the highest level of Y783 residue phosphorylation occurring at 2 min (Figure 5(a)). Preincubation of ECV304 cells with PTX or apocynin prevents PLC-*γ*1 (Y783) phosphorylation (Figure 5(b)), suggesting that PLC-*γ*1 activation depends on FPR1 stimulation and NADPH oxidase-dependent ROS generation.

PLC-*γ*1 catalyzes the hydrolysis of the phosphatidylinositol (4,5)-bisphosphate (PIP2), which results in the production of diacylglycerol (DAG) and inositol 1,4,5-trisphosphate (IP3). IP3 triggers the release of calcium from endoplasmic reticulum and, therefore, an increase in its intracellular concentration, whereas DAG activates protein kinase C (PKC). The PKC isoenzymes PKC*α*, PKC*β*, and PKC*ζ* are implicated in VEGF-mediated signalling [41, 56]. In N-fMLP-stimulated ECV304 cells, we investigated the activation of PKC isoenzymes by analyzing their membrane translocation. We observed that in response to the FPR1 agonist, PKC*α*, PKC*β*ΙΙ, and PKC*ζ* translocate to the membrane and a substantial increment in their amount was found within 2 min of N-fMLP treatment. On the other hand, we did not observe PKC*δ* translocation (Figure 5(c)).

### 3.6. FPR1 Stimulation by N-fMLP Promotes Wound Healing and Capillary-Like Network Formation

Endothelial cell migration is a very critical event during the angiogenesis process. In tumor angiogenesis, endothelial cells invade the surrounding basement membrane and migrate into the stroma. Finally, they organize themselves in the formation of new blood capillaries, which are crucial for tumor growth.

**Figure 6:**
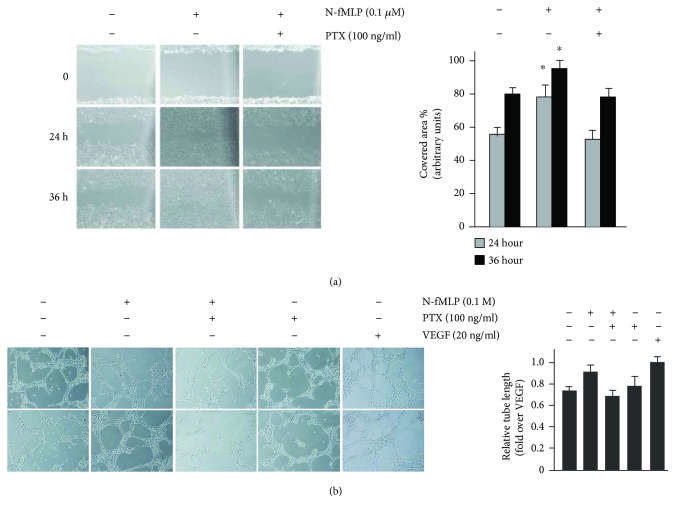
Wound healing and capillary-like network formation induced by FPR1. (a) Representative images (left) and bar graph quantification (right) of ECV304 cell migration from 5 independent experiments. Cells were incubated with 0.1 *μ*M N-fMLP or vehicle in the presence or absence of PTX. Images were acquired at different times (0, 24, and 36 hours) after wound injury (scale bar: 20 *μ*m). (b) Capillary-like network formation (left) was performed in Matrigel-coated plates. ECV304 cells were incubated with N-fMLP in the presence or absence of PTX, and bar graphs (right) show the quantification of relative tube length from four independent experiments. Cells incubated with VEGF (20 *μ*g/ml) were used as a positive control (scale bar: 50 *μ*m). ^∗^*p* < 0.05 compared with unstimulated cells.

Several signalling cascades are implicated in Flk-1/KDR-mediated migration. These involve Y1175 residue phosphorylation and, in turn, the activation of PI3K, as well as the phosphorylation of the Y951 residue, which is a binding site for TSAd [41]. To assess whether FPR1 stimulation by N-fMLP induces cell migration, thus promoting wound closure, we tested ECV304 cells in an *in vitro* wound healing assay. Our results show that N-fMLP induces a more rapid cell migration with respect to unstimulated cells, after both 24 and 36 hours (Figure 6(a)). The preincubation with PTX, before stimulation, prevents N-fMLP-induced wound closure (Figure 6(a)), suggesting that it depends on FPR1 activation.

We also assessed the effects of N-fMLP on capillary-like network formation in a Matrigel assay, which is considered an *in vitro* correlate of angiogenesis. The level of capillary-like network formation was analyzed by measuring the tube length after 1 day of culture. Cells incubated with 20 ng/ml VEGF were used as a control. As shown in Figure 6(b), FPR1 stimulation by N-fMLP results in a significant increase in capillary-like network formation, which is prevented by preincubation with PTX. The tube lengths were substantially increased also in control ECV304 cells exposed to VEGF, compared to untreated cells (Figure 6(b)).

## 4. Conclusions

We demonstrate that, in ECV304 cells, FPR1 stimulation by N-fMLP results in NADPH oxidase-dependent ROS production and VEGFR2 transphosphorylation. Moreover, we demonstrate that ROS bridge the signals from FPR1 to Flk-1/KDR, as evidenced by the results obtained with apocynin and with p22^phox^ silencing on VEGFR2 transactivation and on the intracellular signalling cascades elicited by this receptor. We also show that, as a result of the transactivation mechanism, phosphotyrosines Y951, Y996, and Y1175 of VEGFR2 create anchoring sites for the enrollment and activation of the PI3K/Akt and PLC-*γ*/PKC pathways, fostering some of the molecular responses elicited by VEGFA. Finally, we prove that the FPR1-induced signalling promotes cellular migration and capillary-like network formation of ECV304 cells.

GPCRs represent the largest family of drug targets, which can bind to receptors with high selectivity and regulate several functions in a predictable manner. The observation that each GPCR can engage multiple signalling partners, driving multiple cellular responses, leads to the concept that different ligands can have distinct efficacies toward these different pathways.

GPCRs can trigger signalling cascades in a ligand-specific manner and can cross-talk with RTKs by amplifying intracellular signalling pathways. NADPH oxidase-derived ROS act as signalling molecules by reversible oxidative inactivation of cysteine sulfhydryl groups of PTPs, which can, in turn, control the activity of RTKs and their transactivation [19, 20, 24]. Phosphorylation of cytosolic subunits p47^phox^ and p67^phox^ is required for NADPH oxidase activation. Our results show that ROS generation by NADPH oxidase is tightly regulated and depends on FPR1 stimulation by N-fMLP, which triggers p47^phox^ phosphorylation and, in turn, superoxide generation. The finding that ROS mediate Flk-1/KDR transactivation, playing a crucial role in VEGFR2 signalling related to angiogenesis, provides new insights into NADPH oxidase and/or FPR1 as possible targets for therapies against angiogenesis-dependent diseases. The identified antiangiogenic drugs targeting the VEGFR2 signalling pathways are shared by several RTKs that do not evoke angiogenesis, and the actual antiangiogenic therapies, which target either VEGFA action or Flk-1/KDR activity, may induce the upregulation of other RTKs to overcome the VEGFR blockade. Cross-talk between FPR1 and Flk-1/KDR provides further opportunities for drug discovery strategies for angiogenesis driven by an increase in VEGFR2 activity, disputing actual thinking in the notion of pharmacological targets. Elucidation of the signalling cascades responsible for VEGFR2 transactivation can contribute to the identification of new therapeutic targets able to interfere with the FPR1 pathway. Furthermore, our results suggest that targeting both FPR1 and VEGFR2 might provide improved therapeutic effects, compared to targeting either receptor distinctively.

## Figures and Tables

**Table 1:** Forward and reverse PCR primer sequences.

Primers	Primer sequence	Product size
*β*-Actin	5′-TGATCACCATTGGGAATGAG-3′	154 bp
	5′-CAGTGTGTTGGCGTAGAGGT-3′	
Flk-1/KDR	5′-GAGGGCCACTCATGGTGATTG-3′	709 bp
	5′-TGCCAGCAGTCCAGCATGGTCTG-3′	
Flt-1	5′-GAGAATTCACTATGGAAGATCTGATTTCTTACAGT-3′	498 bp
	5′-GAGCATGCGGATAAATACACATGTGCTTCTAG-3′	
Flt-4	5′-CCCACGCAGACATCAAGACG-3′	380 bp
	5′-TGCAGAACTCCACGATCACC-3′	
FPR1	5′-GACCACAGCTGGTGAACAGT-3′	474 bp
	5′-GATGCAGGACGCAAACACAG-3′	
FPR2	5′-GGATTTGCACCCACTGCATTT-3′	528 bp
	5′-ATCCAAGGTCCGACGATCAC-3′	
FPR3	5′-GAGTTGCTCCACAGGAATCCA-3′	760 bp
	5′-ATAGGCACGCTGAAGCCAAT-3′	
p47^phox^	5′-GGTGGGTCATCAGGAAAGAC-3′	210 bp
	5′-GCAGAAAACGGACGCTGTTG-3′	
p22^phox^	5′-TGTGCCTGCTGGAGTACCCC-3′	441 bp
	5′-ACACGACCTCGTCGGTCACC-3′	
p67^phox^	5′-GCCAGGTGAAAAACTACTGC-3′	246 bp
	5′-CTTCCAGCCATTCTTCATTC-3′	
